# Isolated annuloplasty in elderly patients with secondary mitral valve regurgitation: short- and long-term outcomes with a less invasive approach

**DOI:** 10.3389/fcvm.2023.1193156

**Published:** 2023-10-16

**Authors:** Ulvi Cenk Oezpeker, Daniel Hoefer, Fabian Barbieri, Can Gollmann-Tepekoeylue, Holfeld Johannes, Engler Clemens, Ersahin Suat, Sakic Adel, Rajsic Sasa, Ludwig Mueller, Michael Grimm, Nikolaos Bonaros

**Affiliations:** ^1^ Department of Cardiac Surgery, Medical University of Innsbruck, Innsbruck, Austria; ^2^ Department of Cardiology, Angiology and Intensive Care Medicine, Deutsches Herzzentrum der Charité, Berlin, Germany; ^3^ Department of Cardiology, Angiology and Intensive Care Medicine, Charité—Universitätsmedizin Berlin, Corporate Member of Freie Universität Berlin and Humboldt- Universität zu Berlin, Berlin, Germany; ^4^ Department of Cardiovascular Surgery, Sakarya University, Adapazari, Türkiye; ^5^ Department of Anesthesiology and Intensive Care Medicine, Medical University of Innsbruck, Innsbruck, Austria

**Keywords:** secondary mitral valve regurgitation, elderly, heart failure, annuloplasty, less and minimally invasive mitral valve surgery

## Abstract

**Background:**

Long-term outcomes of elderly and frail patients with secondary mitral valve regurgitation (MR) are inconclusive. Especially in patients with co-morbidities such as atherosclerosis who are suffering from heart failure, optimal medical therapy (OMT) is the preferred therapy relative to surgical or percutaneous interventions. It remains challenging to identify the most successful therapy to improve symptoms and increase life expectancy. To reduce surgical trauma for these patients, minimally invasive mitral valve surgery (MIMVS) was developed; this has shown promising medium-term results, but there is still a lack of evidence regarding long-term results. The aim of this investigation was to describe the long-term outcomes of less invasive mitral valve surgery (MVS) in elderly patients.

**Methods:**

In this longitudinal retrospective analysis, 67 patients (aged ≥70 years) with secondary MR who underwent MV repair ± tricuspid valve repair (TVR) were identified. MVS was performed via minithoracotomy (MT) in most cases (*n* = 54); in patients with contraindications for MIMVS, partial upper sternotomy (PS) was the preferred route for surgical access (*n* = 13). The appropriate access route was chosen according to the patient's clinical condition and comorbidities. We analyzed reoperation-free long-term survival, combined operative success (lack of residual MR, conversion to MV replacement, or larger thoracic incisions), and perioperative safety (at 30 days: mortality, re-thoracotomy, ECMO, pacemaker implantation, dialysis, longer ventilation, stroke, myocardial infarction). In a subgroup analysis, we compared long-term survival in MVS patients with and without TVR.

**Results:**

The median age of patients (62.7% female) was 74 years (interquartile range: 72–76 years), with a median EuroSCORE2 of 2.8% (1.5%–4.6%) and N-terminal pro-brain natriuretic peptide plasma levels of 1,434 ng/L (1035–2149 ng/L). The median follow-up period was 5.6 years (2.7–8.5 years). The reoperation-free long-term survival rate up to 10 years was 66.2%. Combined operative success and perioperative safety were achieved in 94% and 76% of patients, respectively. Additional TVR was performed in 56.7% of patients, without any significant difference in survival rates compared to the group without TVR (*p* = 0.417; HR 1.473, 95% CI 0.578–3.757).

**Conclusion:**

Less invasive MV repair for secondary MR shows excellent operative success and safety in selected patients. Freedom from significant MR and from the need for reoperation indicates long-lasting efficacy. These results should be considered in heart team discussions regarding allocation of patients to surgical mitral procedures.

## Introduction

Secondary mitral regurgitation (MR), with its underlying ischemic or idiopathic cardiomyopathy, is a common valvular dysfunction (1). In the last decade, secondary atrial mitral valve regurgitation has additionally been described as an individual subcategory (2). Establishment of prognosis in these pathologies requires precise differentiation of secondary MV regurgitation etiology and grading of the severity of MR (3–5). The evidence supporting mitral valve surgery (MVS) or percutaneous interventions (6, 7) in patients with secondary mitral valve regurgitation is still under discussion (8, 9). Based on the decision targeting the least harmful therapy, however, selected patients can benefit from either interventional or surgical therapies with acceptable short- and medium-term results. Especially in elderly and often frail patients with multiple co-morbidities, surgical therapeutic options carry risk factors limiting their feasibility only to a highly select subset of patients (10). However, data on long-term results, especially in elderly patients who are refractory to OMT, remain inconclusive, leaving a gray area with a number of unanswered questions (11, 12). Primary or consecutive implantation of long-term mechanical circulatory systems or heart transplantation is not feasible for broad application, or even not possible in patients above 70 years of age (13–15). On the other hand, life expectancy is continuously rising among the elderly population. Such patients have typically been rejected for cardiac surgery, and the only remaining therapeutic option, if found to be suitable, consists of percutaneous interventions. The main goals of all therapies are to reduce interventional trauma to elderly patients, to improve prognosis, and to reduce rates of hospitalization due to heart failure (16, 17).

Several recent investigations have presented promising data on minimally invasive MVS (MIMVS) with access via minithoracotomy (MT) for reduction of surgical trauma (18–20). However, safe MIMVS is only possible in patients without advanced atherosclerosis (21, 22). Moreover, there are several other clinical and anatomical conditions that render MIMVS infeasible (23–25). In these cases, partial upper sternotomy (PS) could be a valid alternative for less invasive surgical access to keep operative trauma as low as possible. However, data on MIMVS and the less invasive PS in elderly patients are rare. The aim of this investigation was to present data on elderly patients (aged >70 years) with secondary mitral valve regurgitation who were operated either via MT access or via PS access.

## Patients and methods

### Patients

Among 1,534 patients who underwent operation on the MV for the first time via MT or PS access between March 2001 and February 2021, 387 patients aged ≥70 years were identified. Of these, 90 patients suffered from secondary MV regurgitation. From this cohort, 15 patients were excluded from the PS access group due to having additionally undergone aortic valve replacement. In addition, five elderly patients were excluded from the PS access group due to having undergone additional CABG with venous grafts to the RCA (*n* = 4) or the proximal LAD (*n* = 1). Finally, another three patients were also excluded as they had received direct MV replacement. Ultimately, data from 67 patients who had undergone isolated MV repair or MV repair combined with TV repair ± atrial ablation were entered into this investigation. Patients were also analyzed for MR etiology [secondary to ischemic or idiopathic ventricular dilatation or to atrial dilatation (secondary atrial MR)].

In 54 patients, the preferred access was via MT, while the PS approach was employed in 13 patients. The appropriate access route was selected according to several criteria following institutional protocols. Specifically, the anatomical or clinical conditions under which patients were considered unsuitable for safe MIMVS were: severely atherosclerotic descending aorta or femoral arteries not amenable for peripheral cannulation (*n* = 5); severe pulmonary hypertension (*n* = 4); aortic valve regurgitation with a possible risk of aortic valve replacement (*n* = 2); and severely impaired left and/or right ventricular function (*n* = 1). Moreover, if the patient was found to be generally in poor clinical condition (*n* = 1), PS was performed instead of MT. To aid decision-making, all patients underwent a computerized tomography scan preoperatively.

### Surgical procedures

Both operative techniques have been described in detail in previous publications (26, 27). Briefly, using the PS access route, we started at the sternal notch with extension of the incision into the left fourth intercostal space. Cannulation for cardiopulmonary bypass (CPB) was performed directly via the ascending aorta and the superior vena cava. For drainage of the inferior vena cava (VC), the femoral vein was cannulated percutaneously using the Seldinger technique. Correct positioning of the femoral cannula was achieved after establishing CPB, and the superior and inferior VC were snared in order to prevent airlock. After opening of the right atrium, the interatrial septum was incised at the level of the fossa ovalis with extension into the left atrial roof to achieve optimal surgical exposure.

For the MT access route, cardiopulmonary bypass was installed via femoro-femoral cannulation with an additional 5 Fr distal leg perfusion cannula to avoid perfusion deficiencies. In patients with increased body surface area, or in cases of planned TVR, an additional venous cannula was inserted into the right jugular. The MT was performed in the fourth intercostal space. Depending on patient sex and build, a periareolar or a 3–4 cm-long skin incision lateral to the nipple or a similar incision in the submammary fold was performed. The third intercostal space on the anterior axillary line was used for the endoscope and the Chitwood clamp. A soft tissue retractor wound protector was used, avoiding rib spreading.

Semi-rigid annuloplasty rings were used in all procedures. In 60 patients (89.6%), Physio 2 annuloplasty rings were used; in three patients, the Livanova Memo 3D was used; and in two patients, the SJM RSAR ring was implanted. In one patient, a posterior band annuloplasty was performed and one IMR annuloplasty ring was used. We performed restricted annuloplasty using rings one size smaller than usually measured on the anterior leaflet (overcorrection). In three patients, additional repositioning of both papillary muscles after primary MV repair failure was necessary. A tricuspid valve repair was performed with semi-rigid rings in patients with severe tricuspid valve regurgitation or annular dilatation more than 21 mm/m^2^ BSA, regardless of type of access.

### Study design and data acquisition

The data for this retrospective cohort study were obtained from the institutional MVS database. Survival data were acquired from the national death registry of Austria (Statistik Austria), and patients without an event were censored at the end of the follow-up period. Written informed consent for the scientific use of clinical data was obtained from all patients as part of the quality control program of the Medical University of Innsbruck, which was approved by the local ethics committee (13 February 2020; EC Nr.: 1203/2019) and the Austrian Ministry of Health. The investigation complied with the principles outlined in the Declaration of Helsinki. Follow-up was conducted via outpatient visits as well as phone calls to the patients and their attending physicians, who sent us the echocardiographic and ECG findings. Almost all patients underwent a TTE provided by their hospital, rehabilitation center, or cardiologist after six months (97.01%; two patients expired within 6 months) and then every year postoperatively. Some patients missed the yearly TTE; in these cases, we report the next one.

### Statistical analysis

Continuous variables are expressed in the form of medians with the interquartile range; categorical variables are reported in the form of numbers and percentages. Kaplan–Meier curves were created for visualization of time-related events during follow-up, and differences between groups were assessed using the log-rank test. Statistical analysis was performed using IBM SPSS, version 25 (IBM Corporation, Armonk, NY, USA). *P*-values of 0.05 or less were considered statistically significant.

### Definitions

#### Preoperative parameters

Coronary artery disease was defined as previous PTCA and/or existing 40%–50% stenosis of one or more vessels. Heart failure, with or without preserved left ventricular ejection fraction, was diagnosed according to the guidelines of the American Heart Association (28).

### Primary outcome

The primary endpoint was defined as reoperation-free survival. Specifically, this was defined as freedom from death from any cause and/or reoperation during follow-up due to valve-related complications.

### Secondary outcomes

Secondary outcome parameters within the first 30 days were defined as composite endpoints. First, we assessed operative success, which was defined as freedom from death, and successful primary MV repair without conversion to replacement or to full sternotomy, or residual mitral regurgitation ≤mild. Another secondary outcome parameter was perioperative safety, which was defined as freedom from perioperative myocardial infarction (according to the fourth universal definition of myocardial infarction), stroke, extracorporeal membrane oxygenation (ECMO) support, renal failure necessitating dialysis, permanent pacemaker implantation, mechanical ventilation >24 h, and reoperation for any reason (including bleeding).

## Results

The baseline characteristics and intraoperative parameters of the study cohort are displayed in Tables 1, 2, respectively. The patient cohort was mainly female (*n* = 42, 62.7%), and the median age was 74 years. Twenty-four patients (35.8%) were suffering from reduced left ventricular ejection fraction (≤ 45%) with NYHA classes III and IV. In 16.4% of patients (*n* = 11), atrial dilatation with secondary mitral regurgitation was diagnosed. MIMVS via MT was performed in 80.6% (*n* = 54), while 19.4% of patients (*n* = 13) were operated via use of the less invasive PS. Additional TVR was conducted in 56.7% of patients (*n* = 38). Concomitant endocardial ablation procedures were performed in 28.4% (*n* = 19), and left atrium appendage occlusion in 41.8% of patients (*n* = 28). Conversion to FS due to an inaccessible bleeding site was necessary in two patients. Conversion due to limitation of the surgical field by MT or PS access was not observed see Table 3.

**Table 1:** Preoperative baseline data.

Parameters	Patients with secondary MV regurgitation (*n* = 67)
Demographics	
Age (years)^a^	74 (72–76)
Gender, female (%, *n*)	62.7 (42)
BMI (kg/m²)	24.8 (22.50–26.60)
Cardiac diagnosis	
Idiopathic cardiomyopathy (%, *n*)	77.6 (52)
Atrial functional MR (%, *n*)	16.4 (11)
Ischemic cardiomyopathy (%, *n*)	6.0 (4)
LV-EF (%)^a^	55 (42–61)
HF with preserved EF (%, *n*)	64.2 (43)
Atrial fibrillation (%, *n*)	67.2 (45)
p-Afib (%, *n*)	16.4 (11)
i-Afib (%, *n*)	50.7 (34)
Pacemaker/ICD implantation (%, *n*)	0
Cardiac decompensations (%, *n*)	9.0 (6)
NYHA 3 + 4 (%, *n*)	43.3 (29)
Arterial hypertension (%, *n*)	62.7 (42)
COPD (≥ GOLD 2) (%, *n*)	9.0 (6)
PVD (%, *n*)	4.5 (3)
On dialysis (%, *n*)	0
Active smoking (%, *n*)	4.5 (3)
Hyperlipidemia (%, *n*)	37.3 (25)
History of stroke (%, *n*)	6.0 (4)
PAP systolic ≥50 mmHg (%, *n*)	10.4 (7)
CAD (%, *n*)	6.0 (4)
EuroSCORE 2^a^	2.76 (1.50–4.60)
Creatinin (mg/dl)^a^	0.95 (0.82–1.08)
NTproBNP (ng/L)^a^	1,434 (1035–2149)

Afib, atrial fibrillation; CAD, coronary artery disease; COPD, chronic obstructive pulmonary disease; i-Afib, intermittent atrial fibrillation; LV-EF, left ventricular ejection fraction; NYHA, New York Heart Association; PAP, pulmonary arterial pressure; p-Afib, permanent atrial fibrillation; PVD, peripheral vascular disease.

^a^ Data are presented in the form of median and interquartile range.

**Table 2:** Preoperative echocardiographic parameters.

Parameters	MVS (*n* = 67)
LV-EF (%)^a^	55 (42–61)
LVEDD (mm)^a^	54 (46–59)
LV-dilation (LVEDD >55 mm) (%, *n*)	34.3 (23)
Left atrium dilatation (> 40 mm) (%, *n*)	85.1 (57)
MI grade moderate-to-severe (%, *n*)	77.6 (52)
MI grade severe (%, *n*)	20.9 (15)
Multiple MV-regurgitation jets (%, *n*)	10.4 (7)
LV-dyskinesis (%, *n*)	34.3 (23)

LV, left ventricular; LVEDD, left ventricular end-diastolic diameter; LV-EF, left ventricular ejection fraction; MI, mitral insufficiency; MV, mitral valve.

^a^ Data are presented in the form of median and interquartile range.

**Table 3:** Intraoperative parameters.

Parameters	MVS (*n* = 67)
Minithoracotomy access (%, *n*)	80.6 (54)
Partial upper sternotomy (%, *n*)	19.4 (13)
MV repair with annuloplasty ring (%, *n*)	100 (67)
Plus TVR (%, *n*)	56.7 (38)
Additional ablation (%, *n*)	28.4 (19)
LAA-closure (%, *n*)	41.8 (28)
CPB time (min)^a^	173 (134–220)
Aortic cross-clamp time (min)^a^	87 (71–111)
Conversion to FS (intraoperative) (%, *n*)	3.0 (2)

CBP, cardiopulmonary bypass; ECMO, extracorporeal membrane oxygenation; LAA, left atrial appendage; MVS, mitral valve surgery; TVR, tricuspid valve repair.

^a^ Data are presented in the form of median and interquartile range.

### Endpoints

**Figure 1:**
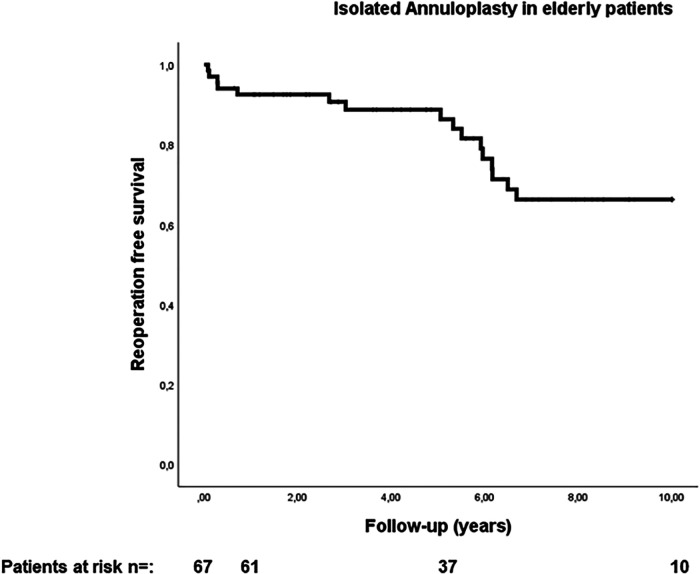
Reoperation free survival in elderly patients with secondary mitral valve pathology and isolated annuloplasty.

**Figure 2:**
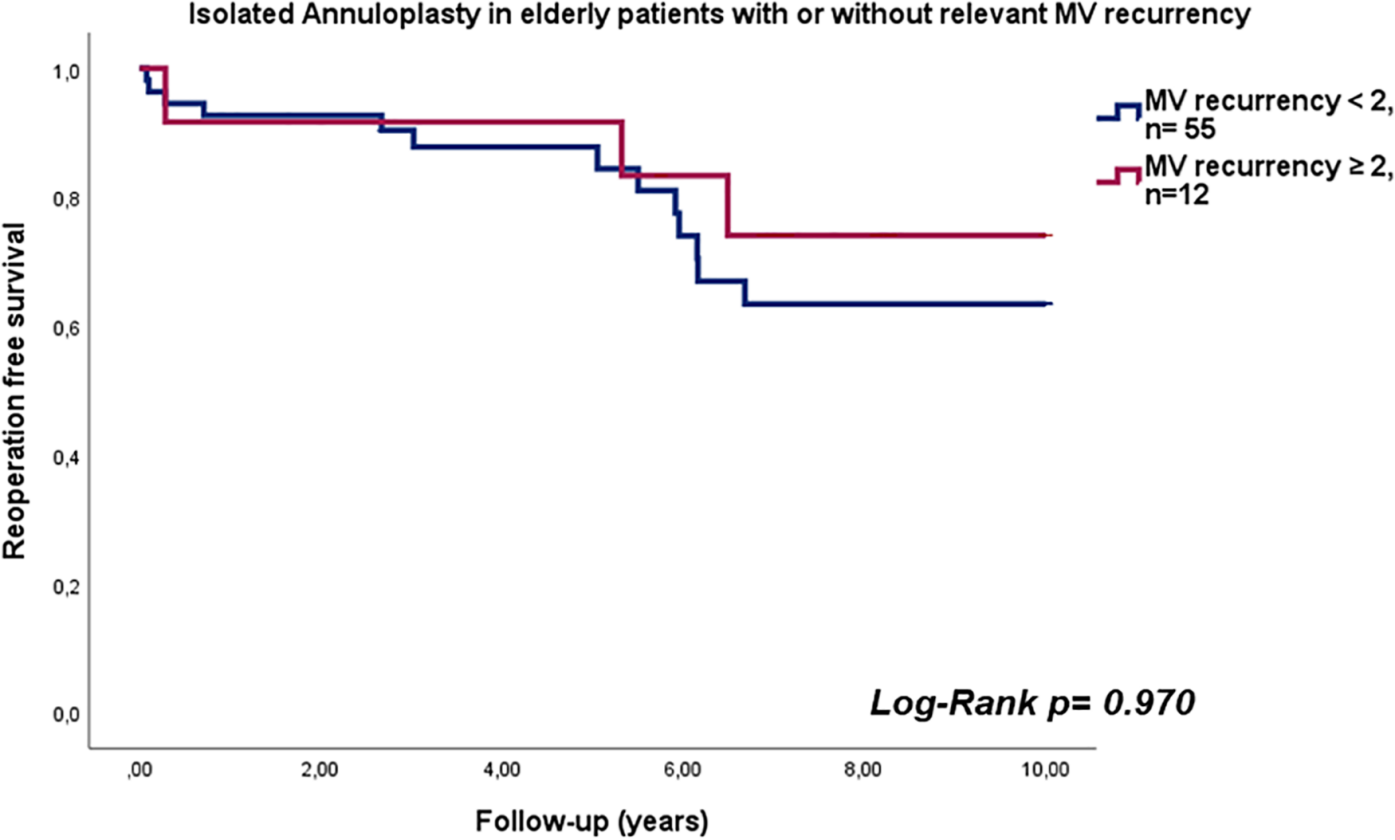
Reoperation free survival in elderly patients with isolated annuloplasty with or without relevant mitral valve recurrency.

The median duration of follow-up was 5.6 years (interquartile range: 2.7–8.5 years). Estimated reoperation-free long-term survival at 10 years in this study was 66.2% (Figure 1). One patient expired within 30 days after surgery and four patients (6%) expired within one year. Heart failure was the cause of death in 53% of cases. In addition, further 10% of deaths were attributed to cerebral stroke, which were cardiac-associated. In 21% of patients, the causes of death were non-cardiac, with 21% of these being attributed to carcinoma. All other patients died of combined cardiac and non-cardiac causes. Unfortunately, no pathohistological investigation or autopsy was performed in the cases of 93% of patient deaths due to advanced age being the defining cause of death. Twelve patients in our cohort (17.9%) developed MV recurrence ≥2 (moderate MV recurrence, *n* = 8; moderate-to-severe, *n* = 3; severe, *n* = 1) during follow-up. There was no difference in survival between patients who developed MR ≥2 during follow-up and those who did not (*p* = 0.970; HR 0.972, 95% CI 0.222–4.249; see Figure 2). However, the patient with severe MV recurrence was rejected for reoperation due to advanced age and co-morbidities.

### Secondary outcomes

Combined operative success was achieved in 94% of patients (*n* = 63). Conversion to FS was necessary in 3% of cases (*n* = 2), while residual MV regurgitation ≥moderate was additionally recognized in two patients, resulting in additional cross-clamping for correction of the result. In addition, two patients who underwent the PS approach needed conversion to FS due to uncontrollable bleeding and pericarditis with tight adhesions (Table 4).

**Table 4:** Secondary outcomes.

Parameters	MVS (*n* = 67)
Combined operative success (%, *n*)	94.0 (63)
Combined perioperative safety (%, *n*)	76.1 (51)
30-day mortality (%, *n*)	1.5 (1)
1-year mortality (%, *n*)	6.0 (4)
Early (30-day) failure of MV repair (≥ 2) (%, *n*)	1.5 (1)
MV regurgitation ≥trace <moderate during follow-up (%, *n*)	28.4 (19)
MV regurgitation >moderate during follow-up (%, *n*)	1.5 (1)

MV, mitral valve; MVS, mitral valve surgery.

Combined (30-day) perioperative safety was achieved in 76.1% of patients (*n* = 51). In 11.9% of patients (*n* = 8), postoperative ventilation was necessary for longer than 24 h. Low output syndrome with inotropic support was observed in 6% (*n* = 4), with the need for ECMO support in 3% of patients (*n* = 2). Postoperative bleeding requiring re-thoracotomy was seen in 7.5% of patients (*n* = 5). In two patients, the bleeding concerned the thoracotomy area; in two, the ports; and in one patient, the right atrium. Acute renal failure with new onset of renal dialysis was necessary in 6% of patients (*n* = 4). Finally, perioperative myocardial infarction was detected in one patient (Table 5).

**Table 5:** Postoperative outcomes.

Parameters	MVS (*n* = 67)
Length of ICU stay (days)^a^	1 (1–2)
Ventilation >24 h (%, *n*)	11.9 (8)
ECMO (%, *n*)	3.0 (2)
Low-output syndrome (%, *n*)	6 (4)
Re-exploration due to post-op bleeding (%, *n*)	7.5 (5)
Dialysis (new) (%, *n*)	6.0 (4)
Pacemaker implantation (%, *n*)	3.0 (2)
Red blood cell units (%, *n*)	1 (0–3)
Stroke (hemorrhagic/embolic) (%, *n*)	0
Myocardial infarction (%, *n*)	1.5 (1)
Sternal/thoracic deep wound infection (%, *n*)	0
Groin wound infection (%, *n*)	1.5 (1)
Gastrointestinal complications (%, *n*)	0
Length of hospital stay (days)^a^	8 (7–10)

ICU, intensive care unit; MV, mitral valve; MVS, mitral valve surgery; post-op, postoperative.

^a^ Data are presented in the form of median and interquartile range.

### Subgroup analysis

**Figure 3:**
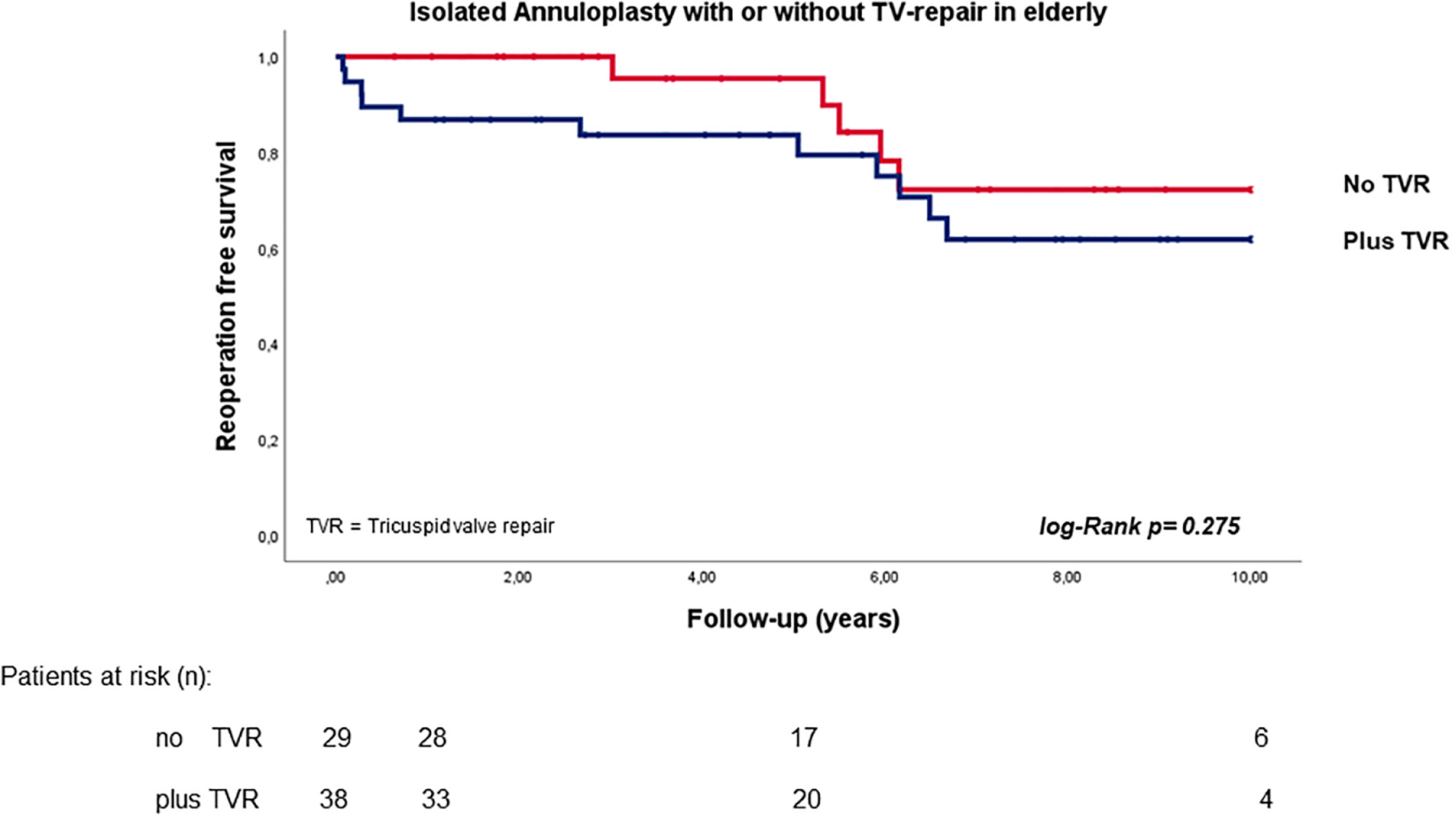
Reoperation free survival in elderly patients with secondary mitral valve pathology and isolated annuloplasty with or without tricuspid valve repair.

In a subgroup analysis, the log-rank test showed comparable reoperation-free survival in elderly patients regardless of whether TV repair was performed or not (*p* = 0.417; HR 1.473, 95% CI 0.578–3.757; see Figure 3).

## Discussion

MVS for secondary MV regurgitation is a gray area with low levels of supporting evidence (29), making OMT and/or percutaneous interventions the preferred therapy in these patients. The lack of evidence supporting MVS is exacerbated in elderly patients, as their co-morbidities and frailty lead to percutaneous interventions being the preferred option in even more cases. To offer these patients the optimal care with long-lasting control of MV regurgitation, reduction of the trauma of cardiac surgical procedures is mandatory (10).

Several studies have described acceptable long-term results for MVS in patients with secondary MV regurgitation. However, data on the outcomes of isolated annuloplasty in elderly patients remain rare, leaving many unanswered questions regarding the most suitable surgical treatment. Our data indicate a 10-year reoperation-free survival rate of 66% following the use of a less invasive access route, which is comparable to regular life expectancy among the age- and sex-matched healthy population (30).

The use of less invasive forms of access reduces operative trauma, resulting in earlier mobilization of patients and thus favoring recovery (31). However, combined operative suc-cess was achieved in only 94% of patients. The reason for this reduced operative success rate was the cases of conversion to FS. This complication is comparable with those occurring in previously described investigations using this access route (27, 32). Another reason for the reduced operative success rate was the need for second cross-clamping due to MV repair failure without switching to MV replacement in the MT group. In the two patients in question, an additionally papillary muscle sling was performed to allow acceptable MV competency. This procedure was additionally performed in one patient after the water test showed a highly insufficient MV.

The preferred access route, if available, should be MIMVS via MT in selected patients, keeping operative trauma as low as possible (20, 33). However, some comorbidities (such as peripheral or aortic atherosclerosis, pulmonary hypertension, and aortic regurgitation >mild) are considered to be contraindications for MIMVS. These clinical conditions do occur in daily surgical life, and they are, in particular, found at higher frequencies in elderly patients. A complementary, less invasive form of access is PS, which has shown similar long-term results to the MIMVS approach in several studies (32, 33). However, comparison of these two approaches is very difficult, as the influence of selection bias in the PS group cannot be ruled out in these observational investigations. It must also be pointed out that we follow the algorithm of less invasiveness, with the result being that we perform full sternotomy for MV surgery without CABG in only one or two cases per year. In addition, the PS approach may have a higher incidence of postoperative permanent pacemaker implantations due to injury of the sinus node artery as a result of dissecting the left atrial roof in the course of using this approach for adequate surgical exposure. This potential complication, however, has been discussed controversially in several publications (34, 35). In fact, two patients in our study, both in the PS access group, received a permanent pacemaker.

Combined perioperative safety was achieved in 76% of all patients. The main reason for this reduced success rate was the prolonged ventilation duration in eight patients. This is in accordance with several publications with elderly patients, in whom the prevalence of requiring a longer period of mechanical ventilation is higher (36, 37). In addition, the higher incidence of postoperative delirium in elderly patients (up to 50%) is an independent risk factor for prolonged ICU stay among these patients (38). Despite this, ICU stays were short in our investigation; nevertheless, the median hospital stay was eight days (IQR: 7–10). It must be pointed out that the median length of stay among all cardiac surgery patients after surgery in our department is six days, indicating that stays were notably longer in this specific cohort. Indeed, all factors such as ECMO, dialysis, and prolonged ventilation have an impact on hospital stay, prolonging hospital stay duration. This results in a hospital stay >10 days for 25% of patients, representing those patients who have experienced the aforementioned complications.

Additionally, 6% of patients needed postoperative dialysis. The reason for acute renal failure was postoperative cardiac low output syndrome in all of these patients. However, the prevalence was low compared to that reported in *Carrascal* et al. (39). The reason might be that the partial integrity of the pericardium under both approaches and the reconstruction of it in the case of MT access might protect the patient from right heart failure. However, half of these patients underwent ECMO within the first 24 h postoperatively, which still represents a low prevalence.

In fact, the pathogenesis and extent of the secondary MV regurgitation and ventricular dilatation progress are crucial factors in identifying the most suitable therapy (3, 40). Different surgical techniques are described for secondary ventricular and for atrial mitral regurgitation. Several studies have reported that, in secondary atrial MV regurgitation, isolated annuloplasty is sufficiently effective for MV repair. Only 16.4% of patients in our investigation had secondary atrial regurgitation. Indeed, at the time of surgery (when this pathology was not yet described), we defined the MV dysfunction as idiopathic. However, in ventricular functional MV regurgitation, additional subvalvular techniques for remodeling of ventricular dilatation might be necessary (18, 41). Despite this, only three of the patients in our cohort needed an additional repositioning of both papillary muscles after primary MV repair failure with isolated annuloplasty. Therefore, extensive differentiation of the form of MV regurgitation and the relevant risk factors are of immense importance in selecting a suitable, minimally invasive therapy in addition to OMT, and doing so consequently enables improved long-term prognosis. However, our investigation demonstrated that, in elderly patients with secondary MV pathology, isolated annuloplasty is an acceptable and easy-to-perform MV repair technique with satisfactory results. Moreover, there was no statistically significant difference in long-term survival between elderly patients who underwent additional TV repair (as an independent factor against long-term survival) and those who did not (26). However, the patients with concomitant TVR in our investigation had higher mortality during the first year. The reason might be that this group constitutes a sicker population with secondary TV regurgitation, and these patients were referred for surgery at almost a very late stage of the disease.

Conservative OMT, in combination with cardiac resynchronization therapy if indicated, is the first-line therapy. However, in patients with high surgical risk and in those who are refractory to OMT, percutaneous interventions are considered (42–44). In addition to surgical trauma, the systemic inflammatory response to cardiopulmonary bypass is more pronounced with surgical compared to percutaneous interventions (45). Transcatheter interventions produce acceptable short-term results, but their long-term efficacy is yet to be proven. It must be pointed out that the surgical risk described, which may be overestimated by the EuroSCORE 2 for (minimally invasive) MV repair, was lower in our cohort compared to typical transcatheter edge-to-edge repair patients (46, 47). In addition, patients undergoing percutaneous interventions present a higher incidence of significant MV regurgitation during follow-up (3, 42) compared to our patient cohort. Nevertheless, elderly patients were still referred more often to percutaneous interventions than to surgical therapy due to the operative trauma risk. Alternative percutaneous interventions, beyond transcatheter edge-to-edge repair, are currently limited to indirect annuloplasty devices (Carillon®, Cardiac Dimensions, Kirkland, WA, USA) and transcatheter mitral valve replacement (Tendyne®, Abbott Laboratories, Abbott Park, IL, USA), although several others are under investigation. Another previously available percutaneous therapeutic option was the Cardioband® Mitral annuloplasty system (Edwards Lifesciences, Irvine, CA, USA), but this has lost approval (48), with missing medium-term results once again. Nevertheless, it is essential to compare surgical with interventional therapies in relation to long-term outcomes, and interventions must also be approved based on prospective studies.

### Limitations

One major limitation of this investigation is the allocation of patients to one or the other less invasive approach, potentially creating selection bias. As cases of full sternotomy access were excluded from our investigation, we cannot rule out the possibility that surgery using full sternotomy access in elderly patients would be associated with a higher burden of poorer outcomes.

In the past several years, with the continuous evolution of surgical techniques and growing experience with MIMVS, the relative contraindications for the MT approach have changed, leaving only few indications for PS. In addition, better intensive care treatment strategies and optimized medical treatment may have an impact on better outcomes. One further limitation of this study is that we did not differentiate between secondary atrial and ventricular regurgitation, as the diagnosis of secondary atrial mitral regurgitation was made retrospectively by analysis of pre- and intraoperative findings in MV patients.

Another limitation of this investigation was the low EuroSCORE 2 levels of patients, possibly indicating a highly selected cohort for surgical MV intervention. Further limitations were the retrospective data analysis and the relatively small sample size.

## Conclusion

The presented results demonstrate that isolated annuloplasty via less invasive approaches was able to achieve excellent reoperation-free survival in well-selected elderly patients with probable secondary MV regurgitation. However, in functional ventricular mitral regurgitation, additional subvalvular techniques seem likely to be necessary for acceptable long-term prognosis. Nevertheless, finding the most suitable therapy for these elderly patients remains an interdisciplinary and complex decision-making process.

## Data Availability

The original contributions presented in the study are included in the article/Supplementary Material, further inquiries can be directed to the corresponding author.
